# Low skeletal muscle area as a prognostic marker for chronic obstructive pulmonary disease in elderly patients admitted to ICU

**DOI:** 10.1038/s41598-019-55737-z

**Published:** 2019-12-13

**Authors:** Jiehua Zhi, Qing Shan, Lanyu Liang, Han Liu, Hua Huang

**Affiliations:** 1 Department of Gastroenterology, The Affiliated Hospital of Yangzhou University, Yangzhou University, Yangzhou, China; 2 Department of Gerontology, The Affiliated Hospital of Yangzhou University, Yangzhou University, Yangzhou, China; 3 Department of Radiology, The Affiliated Hospital of Yangzhou University, Yangzhou University, Yangzhou, China

**Keywords:** Skeletal muscle, Prognostic markers

## Abstract

Low L3 skeletal muscle area (SMA), which is assessed on computed tomography (CT) images, has been reported to indicate poor clinical outcomes of patients with acute exacerbation of chronic obstructive pulmonary disease (COPD). The dorsal muscle group area at the T12 vertebral level (T12DMA) was used as an alternative to L3 SMA. This study aimed to investigate whether T12DMA could be used as a predictor of in-hospital mortality and long-term survival in elderly patients with COPD admitted to the intensive care unit (ICU). This single-center retrospective case–control study was performed by analyzing the clinical information and measuring T12DMA on chest CT images of elderly patients with COPD admitted to the ICU between May 2013 and May 2018. This study included 136 patients. The multivariate logistic regression analysis showed that T12DMA, neutrophil–lymphocyte ratio, invasive mechanical ventilation, and systemic steroid therapy were independent risk factors for predicting the hospital mortality. The median survival was significantly higher in the high-T12DMA group (214 days) than in the low-T12DMA group (32 days).

## Introduction

Chronic obstructive pulmonary disease (COPD) represents a major public health problem worldwide due to increasing prevalence, morbidity, and mortality. It was ranked the eighth among the causes of disease burden worldwide measured by disability-adjusted life-years in 2015^1^. It was also the fourth leading cause of years of life lost in China in 2017^2^. The prevalence of COPD was 14% in patients aged ≥65 years, with a fold increase for every 10–year increment of age^3^. COPD often presents in aging patients as a component of multimorbidity. Sarcopenia was one of the common comorbidities^4^. Sarcopenia, which is defined as age-associated loss of muscle mass and strength, is highly prevalent among patients with COPD and is associated with a worse prognostic index^4–6^. The prevalence of sarcopenia was reported to be 24% among patients with COPD based on a cross-sectional study of a Southeast Asian population. It was associated with age and Global Initiative for Chronic Obstructive Pulmonary Disease (GOLD) stage^7^. Patients with COPD worsened by respiratory failure requiring mechanical ventilation (MV) were more likely to be admitted to the intensive care unit (ICU). Sarcopenia was a strong predictor of mortality among patients with COPD or other diseases requiring MV in the ICU^8–11^. Meanwhile, muscle wasting, as a major contributor to ICU-acquired weakness, occurred early and rapidly during the first week of critical illness^12^. It was associated with short-term and long-term mortality, as a driver of long-term functional disability after discharge from the ICU^13–15^. Therefore, the diagnosis of sarcopenia might help in preventing the mortality of these patients.

In 2018, the European Working Group on Sarcopenia in Older People (EWGSOP) revised the diagnostic criteria for sarcopenia, which is now determined by both low muscle quantity and quality. The EWGSOP recommends the area measurement on a single cross-section computed tomography (CT) image at the level of the third lumbar (L3) vertebra as an alternative tool, since this area accurately represents the whole-body muscle. Thus, this method may serve as an alternative tool for assessing muscle quantity^16^. The quantification of L3 skeletal muscle area (SMA) using a single transverse CT slice showed that more than 56% of patients with respiratory failure had comorbidity of sarcopenia^8^. However, abdominal CT is not a routine examination for patients with COPD. Chest CT scanning is a conventional assessment of muscle mass and is easy to acquire for patients with COPD. The examination of T12, T11, and T10 may serve as an alternative strategy when L3 is unavailable^17^. Moreover, the decline in paravertebral muscle size and attenuation at T12 on CT images has been reported to be associated with mortality among patients with hip fracture^18^ and those undergoing liver transplantation^19^ and general and vascular surgery^20^. The relationship between the T12 vertebral level (T12DMA) and the mortality related to the acute exacerbation of COPD (AECOPD) remains unclear.

This study retrospectively investigated clinical parameters, including T12DMA, dorsal muscle group (DMG) density at the T12 vertebral level (T12DMD), and other risk factors, to evaluate whether these parameters might serve as prognostic markers for in-hospital mortality among elderly patients with AECOPD requiring ICU admissions for ventilation support. It also explored whether the level of T12DMA at admission might be a predictor of long-term survival among patients with COPD after ICU admission.

## Materials and Methods

### Population cohort and design

This was a retrospective case–control study conducted by the Affiliated Hospital of Yangzhou University, a Chinese tertiary teaching hospital, between February 2013 and May 2018. Patients diagnosed with AECOPD based on the International Classification of Diseases, Tenth Revision codes, including J44.000, J44.101, and J44.100, were consecutively enrolled. All patients were previously diagnosed with COPD based on the GOLD 2017 recommendations. Patients had dyspnea with or without unconsciousness when admitted to the ICU directly from the emergency department or during hospitalization.

The inclusion criteria were as follows: (1) age ≥60 years; (2) invasive mechanical ventilation (IMV) or noninvasive mechanical ventilation (NIMV) during the ICU stay; and (3) chest CT scan performed at admission or within 48 h after admission. The exclusion criteria were as follows: (1) discharge within 24 h; (2) no adequate imaging within 48 h after admission; (3) patients admitted to the ICU due to other diseases and suffering from comorbidity of COPD; (4) tumor history; (5) an ICU admission history; and (6) more than 90 days of ICU stay.

A total of 136 patients were eligible for this study. They were divided into two groups based on their survival when discharged from the hospital. All enrolled patients were followed up until October 31, 2019.

### Demographic and clinical information

Data on demographic statistics, comorbidities, clinical information, laboratory parameters, and in-hospital mortality were abstracted from the electronic medical records. The death date of patients was acquired from the National Population Information Database.

The exposure variables were collected, including sex, age, smoking status, Charlson Comorbidity Index (CCI)^21^, APACHE II scores, Glasgow Coma Score (GCS), NIMV or IMV, administration route of steroids [systemic corticosteroid (SCS) treatment and non-SCS treatment (treatment with inhaled or oral corticosteroids], hospital mortality. White blood cell (WBC) count, neutrophil count, lymphocyte count, platelet count, pH, PCO_2_, and PaO_2_/FiO_2_, were recorded within 24 h of admission to the ICU. Neutrophil–lymphocyte ratio (NLR) and platelet–lymphocyte ratio (PLR) were calculated within 24 h of admission to the ICU. The serum albumin recorded at admission (Albumin_ad_), the minimum value during hospitalization (Albumin_min_), and the difference between Albuminad and Albuminmin (termed as ΔAlbumin) were measured.

### Management of MV during the ICU stay

The selection of mechanical ventilation procedures was initiated based on a decision made by the staff physician according to the symptoms and pathophysiological components. Hypercapnic respiratory failure with a pH < 7.35 was the major criterion based on which the physician initiated the NIMV procedure. The major criteria of IMV were as follows^22^: (1) severe dyspnea involving accessory muscles and paradoxical abdominal motion; (2) respiratory arrest; (3) loss of consciousness; (4) failure of noninvasive treatment; (5) psychomotor agitation requiring sedation; and (6) hemodynamic instability with a systolic blood pressure less than 70 mm Hg or greater than 180 mm Hg.

### Diagnosis of pneumonia

Pneumonia was diagnosed based on the chest CT scan at admission. The CT scan was reviewed by an experienced radiologist. Pulmonary edema, pulmonary fibrosis, tuberculosis, lung cancer, and pulmonary embolism were excluded.

### Measurement of T12DMA and T12DMD

**Figure 1:**
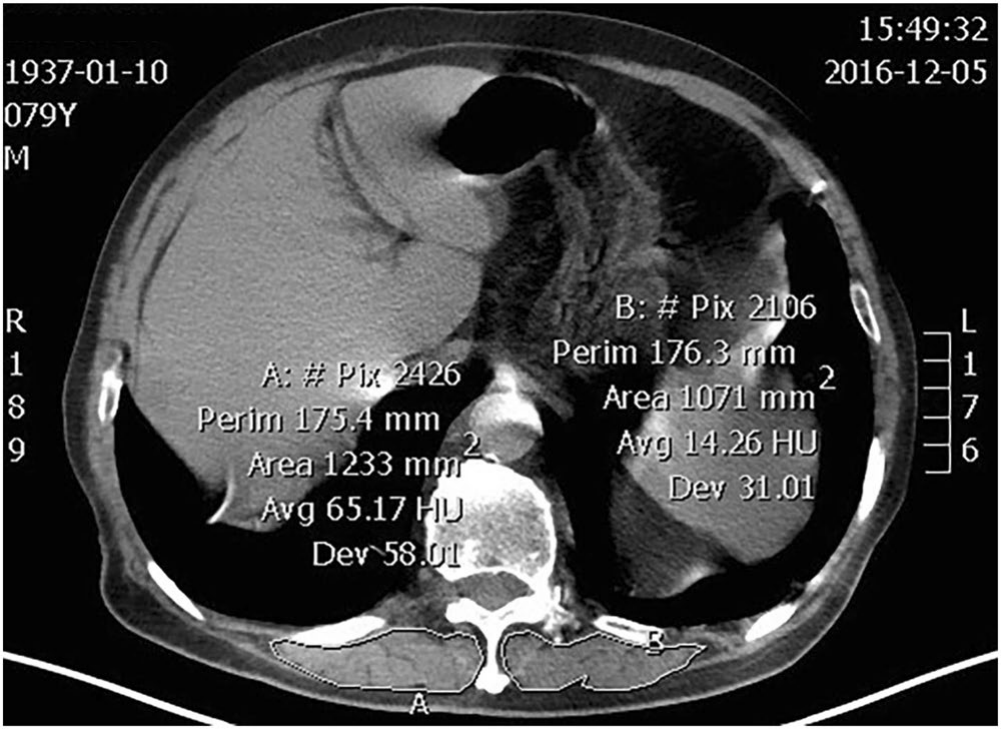
T12DMA of a 79-year-old deceased male patient. The region marked with a solid line indicates the bilateral DMA.

The electronically stored CT images of patients at admission or within 48 h after admission were analyzed using Picture Archiving and Communication Systems (PACS, IMPAX6.3.1.4095, AGFA HealthCare NV, Belgium.). T12DMA was defined as any muscle within the region posterior to the T12 spine and ribs and no more lateral than the lateral-most edges of the erector spinal muscles^19^ (Fig. 1). All imaging analyses were conducted in the cross-sectional area at the level of the 12th thoracic spine and manual sketch ridge of the DMG. The muscle area and density were evaluated using the PACS. T12DMA was recorded as the sum of bilateral DMG area, while T12DMD was recorded as the mean of bilateral DMG radiodensity. The measurements of T12DMA and T12DMD were independently performed by an experienced radiologist and a well-trained doctor, and the final measurements were the average of the two.

### Ethical approval

This study was carried out in accordance with the declaration of Helsinki (2000) of the World Medical Association. This was a retrospective trial. The Human Research Ethics Committee of the Affiliated Hospital of Yangzhou University that approved this study also waived the need for informed consent.

### Statistical analysis

Statistical analyses were performed using SPSS 23.0 (IBM, NY, USA). All demographic and clinical characteristics were compared between the two groups based on the status at discharge (surviving or deceased).

All variables were evaluated for normal distribution using the Shapiro–Wilk test. All quantitative variables in line with the normal distribution were described as mean ± standard deviation. The univariate analysis was performed using an independent-samples *t* test. Variables with non-normal distribution were described as median and interquartile range (IQR). The univariate analysis was performed using the Mann–Whitney *U* test. All qualitative variables were described as number (percentages), and the univariate analysis was performed using the chi-square test.

Pearson coefficients (normally distributed data) or Spearman rho (non-normally distributed data) test were introduced to detect whether T12DMA had any effect on the other laboratory parameters.

Multivariate regression logistic models were constructed to identify the prognostic markers of hospital mortality. The probability of variables entering regression based on the univariate analysis was less than 0.100.

The selected variables were subjected to forward conditional stepwise regression.

To emphasize the prognostic role of T12DMA in hospital mortality, the receiver operating characteristic (ROC) curve was used to analyze the accuracy, sensitivity, specificity, and area under the ROC. The Youden index was obtained by the ROC analysis, and the maximum tangent point was selected to establish the cutoff value. The Kaplan–Meier curves were drawn to illustrate the effect of T12DMA on the long-term survival between the groups based on the T12DMA cutoff value. The significance analysis was conducted using the log-rank test. Two-tailed *P* less than 0.05 was considered to be statistically significant.

## Results

### Demographic and clinical characteristics

A total of 136 patients were enrolled in this study, including 98 male (72.06%) and 38 female (27.94%). The average age was78.00 years (IQR 71.25–83.75). Of the 136 patients, 59 (43.38%) died at discharge. The demographic and clinical characteristics and details of patient samples in the two groups are shown in Table 1. The acute inflammation biomarkers, such as WBC count (9.64, IQR: 7.06–13.76, *P* = 0.026), NLR (12.81, IQR: 5.58–21.71, *P* < 0.001), PLR (201.30, IQR: 127.71–448.65, *P* = 0.006), had significant differences between the groups. T12DMA (23.10, IQR: 19.16–27.25, *P* = 0.005) was lower in the deceased group than in the surviving group (*P* = 0.005). Further, 105 patients (77.20%) had pneumonia at admission, but with no significant differences between the groups. Also, 123 patients (90.4%) who received IMV during ICU hospitalization showed a bad outcome compared with patients who received NIMV (*P* = 0.032). Patients who received SCS treatment had higher in-hospital mortality compared with non-SCS patients (*P* = 0.014).

**Table 1:** Demographic and medical data of the two groups.

Variable	All patients (*n* = 136)	Surviving (*n* = 77)	Deceased (*n* = 59)	Statistic	*P* value
Age (year)	78.00 (71.25–83.75)	77.00 (67.00–83.00)	78.00 (73.00–84.00)	−1.316	0.188
Sex (%)				0.341	0.559
Male	98 (72.06%)	57 (74.00%)	41 (69.50%)		
Female	38 (27.94%)	20 (26.00%)	18 (30.50%)		
Smoking status (%)				2.874	0.238
Current smoking	25 (18.40%)	11 (14.30%)	14 (23.70%)		
Quit smoking	20 (14.70%)	10 (13.00%)	10 (16.90%)		
Never smoking	91 (66.90%)	56 (72.70%)	35 (59.30%)		
CCI scores	2.00 (2.00–3.00)	2.00 (2.00–3.00)	2.00 (2.00–3.00)	−0.094	0.925
GCS	11.00 (6.00–13.00)	11.00 (7.00–14.50)	8.00 (6.00–13.00)	−1.377	0.168
APACHE II	22.00 (20.00–25.00)	22.00 (19.50–26.00)	22.00 (20.00–25.00)	−0.117	0.907
pH	7.27 (7.18–7.36)	7.27 (7.17–7.35)	7.28 (7.18–7.41)	−0.734	0.463
PO_2_ (mm Hg)	75.00 (56.00–100.00)	76.00 (58.00–111.50)	73.00 (54.00–99.00)	−1.407	0.159
PCO_2_ (mm Hg)	78.00 (54.25–106.25)	77.00 (53.50–102.50)	82.00 (55.00–113.00)	−0.646	0.518
PaO_2_/FiO_2_ (mm Hg)	197.06 (137.63–266.25)	209.09 (145.21–276.89)	193.75 (114.00–250.00)	−1.164	0.245
WBC (×10^9^/L)	9.64 (7.06–13.76)	8.67 (6.89–11.33)	11.06 (8.10–15.30)	−2.224	0.026
NLR	12.81 (5.58–21.71)	8.80 (4.46, 18.69)	17.36 (8.68, 28.00)	−3.546	<0.001
PLR	201.30 (127.71–448.65)	180.95 (111.45–346.79)	302.13 (150.77–561.10)	−2.758	0.006
CRP (mg/L)	19.97 (12.64–50.96)	16.28 (11.22–50.90)	30.91 (13.05–52.58)	−1.383	0.167
Albumin_ad_ (g/L)	35.25 ± 4.98	35.44 ± 5.11	35.01 ± 4.84	0.498	0.619
Albumin_min_ (g/L)	28.48 ± 4.14	28.01 ± 3.52	29.09 ± 4.80	1.512	0.133
ΔAlbumin (g/L)	6.77 (3.2–9.25)	6.90 (4.15–9.75)	6.0 (2.1–9.0)	−1.623	0.105
T12 DMA (cm^2^)	23.10 (19.16–27.25)	23.82 (20.38–28.10)	20.59 (16.84–24.95)	−2.799	0.005
T12 DMD (IU)	26.52 (17.79–33.37)	27.05 (18.45–34.80)	24.90 (14.88–30.65)	−1.693	0.091
Pneumonia (%)				0.052	0.820
No	31 (22.80%)	17 (22.10%)	14 (23.70%)		
Yes	105 (77.20%)	60 (77.90%)	45 (76.30%)		
MV (%)				4.587	0.032
IMV	123 (90.40%)	66 (85.70%)	57 (96.60%)		
NIMV	13 (9.60%)	11 (14.30%)	2 (3.40%)		
SCS (%)				6.000	0.014
No	60 (44.10%)	41 (53.20%)	19 (32.20%)		
Yes	76 (55.90%)	36 (46.80%)	40 (67.80%)		

APACHE: Acute physiology and chronic health evaluation.

CCI: Charlson Comorbidity Index.

GCS: Glasgow Coma Score.

MV: Mechanical ventilation.

IMV: Invasive mechanical ventilation.

NIMV: Noninvasive mechanical ventilation.

NLR: Neutrophil–lymphocyte ratio.

PLR: Platelet–lymphocyte ratio.

SCS: Systemic corticosteroid.

T12DMA: Dorsal muscle group area at the T12 vertebral level.

T12DMD: Dorsal muscle group density at the T12 vertebral level.

### Risk factors and survival

As shown in Table 2, the variables in the multivariate regression logistic models were T12DMA [odds ratio (OR) = 0.901; 95% confidence interval (CI), 0.841–0.967, *P* = 0.004], NLR (OR = 1.051; 95% CI, 1.019–1.085, *P* = 0.002), MV (OR = 0.190; 95% CI, 0.036–0.997, *P* = 0.050), and systemic steroid therapy (OR = 2.439; 95% CI, 1.081–5.505, *P* = 0.032). They were independent risk factors for predicting the hospital mortality.

**Table 2:** Logistic regression analysis of the risk factors for in-hospital mortality.

		*B*	SE	Wald	*P*	OR	95% CI	
Step 1^a^	NLR	0.051	0.015	11.667	0.001	1.053	1.022	1.084
	Constant	−1.108	0.295	14.074	0.000	0.330		
Step 2^b^	T12DMA	−0.086	0.034	6.634	0.010	0.917	0.859	0.980
	NLR	0.050	0.015	10.995	0.001	1.051	1.021	1.083
	Constant	0.890	0.802	1.231	0.267	2.435		
Step 3^c^	T12DMA	−0.105	0.036	8.658	0.003	0.901	0.840	0.966
	NLR	0.046	0.015	9.260	0.002	1.047	1.016	1.078
	SCS or not	1.005	0.409	6.031	0.014	2.731	1.225	6.090
	Constant	0.790	0.815	0.942	0.332	2.204		
Step 4^d^	T12DMA	−0.104	0.036	8.485	0.004	0.901	0.841	0.967
	NLR	0.050	0.016	9.754	0.002	1.051	1.019	1.085
	IMV or not	−1.658	0.845	3.853	0.050	0.190	0.036	0.997
	SCS or not	0.892	0.415	4.610	0.032	2.439	1.081	5.505
	Constant	2.568	1.202	4.560	0.033	13.037		

^a^Variable(s) entered on step 1: NLR.

^b^Variable(s) entered on step 2: T12DMA.

^c^Variable(s) entered on step 3: SCS or not.

^d^Variable(s) entered on step 4: IMV or not.

Method: forward conditional stepwise regression; the probability of variable entering regression was 0.05, and the probability of deletion was 0.10.

NLR: Neutrophil–lymphocyte ratio.

T12DMA: Dorsal muscle group area at the T12 vertebral level.

SCS: Systemic corticosteroid.

IMV: Invasive mechanical ventilation.

### Effects of T12DMA on COPD acute exacerbation and long-term survival

T12DMA was associated with the serum albumin level at admission (*r* = 0.230, *P* = 0.007) and the difference between the albumin level at admission and the minimum level of serum albumin during hospitalization (*r* = 0.240, *P* = 0.005) (Table 3).

**Table 3:** Correlation analysis between T12DMA and laboratory parameters.

	r	P
pH	−0.055	0.522
PO_2_ (mm Hg)	0.166	0.054
PCO_2_ (mm Hg)	0.02	0.819
PaO_2_/FiO_2_ (mm Hg)	0.134	0.119
WBC (×10^9^/L)	0.011	0.901
NLR	−0.118	0.17
PLR	−0.117	0.174
CRP (mg/L)	−0.007	0.936
Albumin_ad_ (mg/L)	0.230^**^	0.007
Albumin_min_ (mg/L)	0.106	0.218
ΔAlbumin (mg/L)	0.240^**^	0.005

NLR: Neutrophil–lymphocyte ratio.

PLR: Platelet–lymphocyte ratio.

CRP: C-reactive protein.

Albumin_ad_: The serum albumin at admission, Albumin_min_: The minimum value during hospitalization, ΔAlbumin: Difference between Albumin_ad_ and Albumin_min_ ().

**Figure 2:**
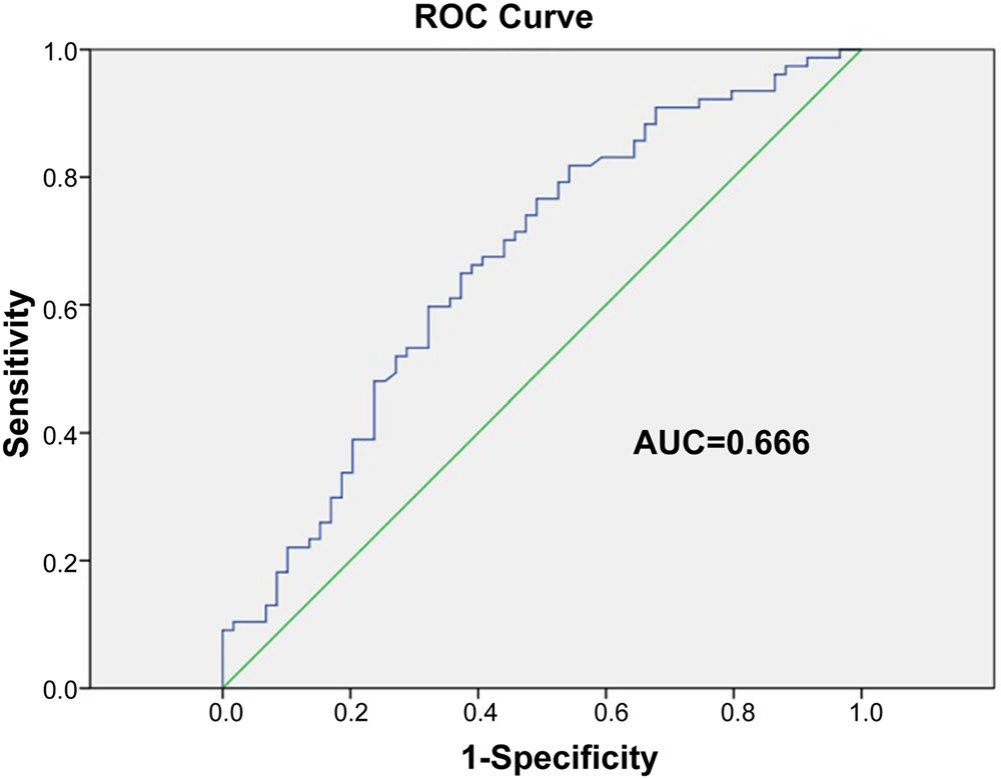
ROC curve of T12DMA to predict the hospital mortality of patients (AUC = 0.666, P = 0.005).

The area under the ROC curve (AUC) for the prognostic role of T12DMA in hospital mortality was 0.666 (*P* = 0.001). The optimal cutoff value of T12DMA to predict survival was 22.515 cm^2^ (Fig. 2).

**Figure 3:**
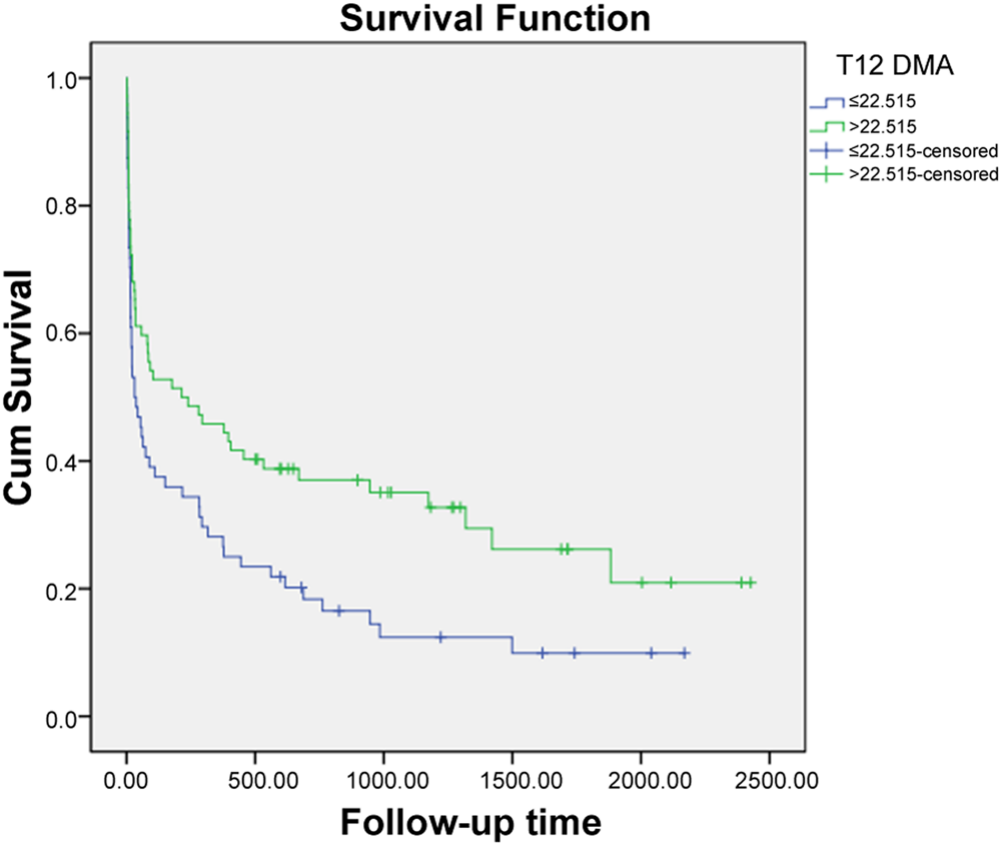
Kaplan–Meier curves of T12DMA to predict long-term survival. The median survival of the high- and low-T12DMAs group was 214 days and 32 days, respectively (P = 0.011).

The relationship between high/low T12DMA groups based on the cutoff value (22.515 cm^2^) and overall survival was displayed in the Kaplan–Meier curves (Fig. 3). The median survival was significantly higher in the high-T12DMA group (214 days) than in the low-T12DMA group (32 days) (*P* = 0.011) (Table 4).

**Table 4:** Log-rank analysis between T12DMA and long-term survival.

	Median survival (d)	*χ*^2^	*P*
≤22.515 (cm^2^)	31	6.454	0.011
>22.515 (cm^2^)	214		

## Discussion

In this study, aging patients with the advanced stage of COPD, who were admitted to the ICU for MV therapy, had a high hospital mortality rate (43.38%). The rates of hospital mortality in patients with COPD vary worldwide, ranging from 11% to 48%^23–25^. T12DMA, inflammation biomarker NLR, IMV, and administered intravenous steroids were independent risk factors for in-hospital mortality among patients with COPD who were admitted to the ICU. As a surrogate of L3 SMA, T12DMA was used to measure the skeletal muscle mass of patients with COPD for diagnosing sarcopenia. This study showed that low T12DMA affected the in-hospital mortality and long-term survival of patients after ICU admission.

Sarcopenia has a high morbidity in patients with stable COPD, indicating a poor prognosis. This problem should not be neglected in patients referred for physical rehabilitation^26,27^. In addition, COPD exacerbation decreased the endurance of skeletal muscles^28^. The present study focused on critical patients with COPD who were admitted to the ICU. In this stage, the low skeletal muscle mass prognosticated high mortality in the acute episode and chronic rehabilitation stage. COPD-related sarcopenia in the stable stage of the disease was associated with age, GOLD stage^7^, and systemic inflammation^27^. However, in this study, T12DMA had no association with the inflammation biomarkers, including CRP, WBC, PLR, and NLR. Low serum albumin concentrations were associated with reduced muscle mass in relatively healthy, well-nourished, elderly men and women^29^. In the present study, T12DMA was associated with the serum albumin level at admission and the loss during hospitalization, but had no relationship with the minimum level of serum albumin. Puthucheary reported that the cross-sectional area of rectus femoris decreased significantly within the first week^12^. Hence, it was hypothesized that critical patients with COPD had skeletal muscle mass loss due to ICU admission and MV therapy. Despite no significant difference between the two groups, the serum albumin level significantly declined in all patients. Muscle mass is maintained by protein homeostasis^30^, with serum albumin being the most relevant. A high skeletal muscle mass at admission may maintain the protein homeostasis for critically ill patients. This hypothesis should be verified in prospective studies. Puthucheary *et al*.^31^ investigated the metabolic phenotype of skeletal muscle wasting in early critical illness, suggesting that non-fat sources and removal of fatty acid supplementation could improve the skeletal muscle energy status.

Further, 50.6% patients had COPD complicated with pneumonia during hospitalization^32^. In the present study, 77.2% patients with COPD, who were admitted to the ICU, had co-existing pneumonia. The majority of AECOPD events were attributed to bacterial or viral infection. This study showed that WBC count, NLR, and PLR, as inflammation biomarkers, were significantly elevated in the deceased group. Especially NLR within 24 h after admission to the ICU was an independent risk factor for predicting the in-hospital mortality. In line with Terradas *et al*.^33^, NLR > 7 was a predictive factor for AECOPD in-hospital mortality. The elevated NLR affected readmission and mortality in the 6-month follow-up period^34^. As demonstrated by Valiollah Amri Malehm, the mortality rate in IMV (54%) was higher than that in NIMV (8%) in an observational cohort study. The mortality rate in IMV and NIMV was 46.34% and 15.38%, respectively. Furthermore, IMV was an independent predictive factor for in-hospital mortality in our study.

Systemic steroid therapy is commonly used for hospitalized patients with AECOPD. This study showed that the intravenous administration of steroids was an independent risk factor for hospital mortality. Bahloul *et al*.^35^ confirmed that systemic corticosteroid therapy could reduce the duration of MV and shorten the ICU LOS. McCann *et al*.^36^ indicated that the prescribed maintenance steroid dose was not associated with the duration of MV or ICU LOS. A meta-analysis performed by Abroug *et al*.^37^ showed no beneficial effect of corticosteroids in critically ill patients. Another open-label study conducted in two ICUs in Tunisia also found negative outcomes^38^. This study provided a negative evidence for aging patients with COPD requiring MV.

This study had several limitations. This was a single-center retrospective study. A certain number of patients were eliminated to analyze the skeletal muscle mass on CT images. Especially very critically ill patients who could not undergo CT often had a bad outcome rapidly within 24 h; they should be excluded from the study according to the study design. Deleting the information on these patients had a minimal effect on the study results. Only 136 patients were enrolled in the study. Insufficient sample size, especially the fewer number of female patients, might have introduced some bias in the results. Hence, large-scale studies with more patients from other centers should be conducted in the future. Besides, this was a retrospective study. Hence, the CT scans after ICU admission were not available for all patients to measure the skeletal muscle mass loss during ICU hospitalization. It was hypothesized that critical patients with COPD had skeletal muscle mass loss during ICU hospitalization according to the sharp decline in serum albumin levels. The acute loss of skeletal muscle mass loss and target nutrition supply among patients with COPD during the episodes of acute exacerbation and ICU admission for MV therapy should be further investigated.

## Conclusions

T12DMA was an independent predictive factor for hospital mortality and a predictor of long-term survival in elderly patients with COPD who were admitted to the ICU. Skeletal muscle mass loss was a crucial problem in the stable and advanced stages of COPD and hence should not be neglected. This study presented an alternative method for measuring the skeletal muscle mass when the CT scans were available, especially for critically ill patients whose body composition like BMI could not be acquired. The clinical parameters, such as NLR, IMV, and systemic steroid therapy, were independent risk factors for predicting the hospital mortality. Large-sample, multi-center studies, involving more female patients, are required in the future to clarify the reasons behind sex-specific difference in findings.

## Data Availability

All data generated or analysed during this study are included in this published article (and its Supplementary Information files).
